# Detect and Repair: Robust Self-Supervised Wearable Sensing Under Missing Modalities

**DOI:** 10.3390/s26082419

**Published:** 2026-04-15

**Authors:** Aboul Hassane Cisse, Shoya Ishimaru

**Affiliations:** Graduate School of Informatics, Osaka Metropolitan University, Osaka 558-8585, Japan; ishimaru@omu.ac.jp

**Keywords:** wearable sensing, multimodal sensor data, missing-modality detection, self-supervised learning, sensor signal reconstruction, physiological signals, IMU data, robust sensing systems

## Abstract

Wearable sensor systems are being increasingly deployed in real-world environments to monitor human activities and cognitive states. However, such systems frequently suffer from degraded or missing sensor modalities due to occlusions, energy constraints, or hardware failures. In this work, we introduce CognifySSL v2.0, a self-supervised learning framework designed to detect and repair missing modalities in real time under simulated real-world missing-modality conditions. The model combines contrastive and masked modeling objectives across multiple physiological and motion signals (e.g., IMU, ECG, EDA) using a fusion architecture with dropout simulation. Evaluation on WESAD demonstrated effective multimodal detection and reconstruction under missing-modality conditions, while experiments on MobiAct validated unimodal robustness and representation learning under sensor dropout. We released our code and interactive visualization dashboard to support reproducibility and future research on robust multimodal fusion.

## 1. Introduction

Wearable sensors are being increasingly deployed in everyday environments to monitor human activities, physiological states, and cognitive workload. These systems typically leverage multiple modalities such as inertial measurement units (IMUs), electrocardiography (ECG), electrodermal activity (EDA), respiration (RESP), and skin temperature (TEMP) to achieve a robust and fine-grained understanding of users’ internal states and behaviors.

However, real-world deployments are notoriously susceptible to missing or corrupted signals. While these challenges are well-documented, evaluating such conditions in fully unconstrained real-world datasets remains limited.

For instance, heart rate data from a smartwatch may be lost due to motion artifacts, poor skin contact, or battery-saving interruptions. Similarly, EDA sensors embedded in wristbands often exhibit signal dropout caused by sweating or reduced skin conductivity. Such issues—arising from occlusion, sensor drift, hardware malfunction, or asynchronous sampling—render many machine learning pipelines brittle, particularly in multimodal settings where complementary signals are jointly exploited. Unlike controlled laboratory environments, real-world wearable deployments routinely experience partial sensing due to battery management, user discomfort, motion artifacts, and environmental interference. In these conditions, discarding samples with missing modalities is impractical and severely limits deployability.

Recent advances in self-supervised learning (SSL) have shown strong potential for learning meaningful representations from unlabeled wearable data, enabling progress in activity recognition and affective computing without manual annotation effort [1,2,3,4]. Nevertheless, most existing SSL-based approaches implicitly assume complete and synchronized modality inputs during both training and inference. This assumption is unrealistic for real-time applications in health, education, and industrial monitoring, where sensor streams are often noisy, delayed, or partially missing.

To address this gap, we propose CognifySSL v2.0, a self-supervised multimodal fusion framework explicitly designed to operate under incomplete sensing conditions. Our approach unifies contrastive learning and masked signal modeling to support robust representation learning while explicitly accounting for sensor unreliability. Unlike prior work that treats missing data implicitly or assumes fixed-modality availability, CognifySSL v2.0 explicitly predicts modality presence, detects missing signals, and reconstructs corrupted modalities at inference time, enabling adaptive and resilient operation under realistic simulated missing-modality conditions in wearable systems.

Compared to existing methods, CroSSL [2] and MU-MAE [3] leverage latent masking strategies, but do not explicitly model modality presence or perform full signal reconstruction at the inference time. COCOA [5] focuses on contrastive alignment across modalities, but lacks mechanisms for repairing missing inputs, while large-scale representation learning approaches such as ImageBind [6] prioritize cross-domain alignment rather than robustness to sensor dropout. In contrast, CognifySSL v2.0 jointly addresses missing-modality detection, signal reconstruction, and deployment feasibility within a unified self-supervised architecture.

We evaluated CognifySSL v2.0 on two publicly available benchmarks: MobiAct [7], an IMU-based dataset for human activity recognition, and WESAD [8], a multimodal dataset for affective state recognition involving physiological and motion signals. The experimental results demonstrated that CognifySSL v2.0 consistently outperformed prior methods, including MU-MAE, CroSSL and COCOA, particularly under controlled modality corruption and ablation scenarios.

In summary, building on the challenges of real-world wearable sensing discussed above, this paper introduces CognifySSL v2.0, a self-supervised framework designed to enable robust multimodal representation learning in the presence of missing sensor data. These controlled dropout scenarios are designed to approximate realistic missing-modality conditions observed in real-world wearable sensing environments, where sensor failure, noise, and occlusion frequently occur.

## 2. Related Work

**Self-Supervised Learning for Human Activity Recognition.** Recent advances in self-supervised learning (SSL) have significantly improved human activity recognition (HAR) by leveraging unlabeled sensor data. Contrastive learning frameworks such as time-contrastive networks (TCNs) [9] and SimCLR-based approaches [10,11] encode temporal dependencies by distinguishing positive and negative signal pairs. Masked modeling strategies have also been adapted from vision to wearable signals, as in TASKED [12] and ST-MAE [13], enabling temporal structure recovery from incomplete observations. However, these methods often assume complete sensor availability during training and inference.

**Multimodal Fusion and Representation Learning.** Multimodal SSL methods aim to align and fuse heterogeneous inputs such as IMU, ECG, EDA, and audio into shared latent representations. Works like MU-MAE [3], CroSSL [2], and ImageBind [6] extend masked autoencoders and contrastive frameworks to multimodal data, enabling cross-modal alignment and transfer. Fusion techniques vary from early/late fusion to attention-based gating [14], but many approaches are not robust to missing modalities. SOAR [15] introduces scalable late-fusion with cross-modal calibration, yet still assumes a predefined fusion architecture and fails under partial modality dropout.

In addition to time-series-specific approaches, large-scale multimodal foundation models such as ImageBind [6] demonstrate scalable joint embedding learning across heterogeneous modalities, including images, audio, text, and IMU data. Trained on billions of aligned data pairs, such models achieve impressive zero-shot generalization across domains. However, they are primarily designed for cross-domain semantic alignment and are not tailored to the temporal dynamics, physiological characteristics, or real-time modality dropout challenges inherent in wearable sensing systems.

**Missing-Modality Imputation and Resilience.** Handling missing or corrupted modalities remains an open challenge. Some methods rely on dropout during training to simulate sensor loss [1], while others use generative recovery, e.g., masked signal reconstruction in RCSMR [16]. Relative contrastive learning [17] improves the robustness to sensor shifts, but does not explicitly detect missing modalities or repair corrupted inputs in real time. Our work introduces a unified framework that (1) detects absent signals, (2) reconstructs missing streams via masked sensor modeling, and (3) maintains discriminative representations via fusion-aware contrastive objectives.

**Benchmark Datasets and Evaluation Gaps.** Despite the emergence of powerful self-supervised learning techniques, few studies have systematically assessed the robustness under modality corruption. To address this gap, we benchmarked our approach on two publicly available datasets that offer complementary evaluation settings: MobiAct [7] and WESAD [8].

MobiAct is a widely used IMU-based dataset for human activity recognition that captures diverse locomotion patterns in daily-life scenarios, making it suitable for evaluating the unimodal robustness and representation quality under sensor dropout. In contrast, WESAD is a rich multimodal dataset for stress and affect recognition, comprising synchronized physiological and motion signals, including electrodermal activity (EDA), which reflects sympathetic nervous system activation; electrocardiogram (ECG) signals used to derive the heart rate and heart rate variability; respiration (RESP), capturing the breathing patterns and rate; skin temperature (TEMP), related to thermoregulation and the stress response; and tri-axial accelerometer (ACC) data describing body motion.

Together, these datasets exhibit varying sampling rates, modality combinations, and ground-truth annotations, making them well-suited for evaluating both unimodal robustness and multimodal resilience under controlled modality corruption. As summarized in Table 1, prior work has rarely examined dynamic modality ablation or real-time sensor dropout, which constitutes a core focus of the proposed CognifySSL v2.0 framework.

## 3. Research Questions and Hypotheses

This study investigated how self-supervised learning can be leveraged to enable robust, real-time inference from incomplete and multimodal wearable sensor streams. We focused on identifying architectural designs, learning objectives, and multimodal fusion mechanisms that explicitly account for sensor unreliability, promote resilience to missing modalities, and preserve the representation quality for downstream human state inference [2,3,15,17,18,19]. Accordingly, this work addresses the following research question: how can a self-supervised architecture detect and repair missing modalities in multimodal time-series data to support robust, efficient, and deployable inference in wearable computing systems?

### 3.1. Sub-Questions

- **RQ1:** How effective is random modality dropout during training in simulating real-world sensor failure scenarios [15,18]?
- **RQ2:** What is the contribution of contrastive alignment vs. masked reconstruction in shaping semantically meaningful latent spaces [3,11,20]?
- **RQ3:** Can the proposed architecture generalize to unseen modality combinations, and how does it perform under varying levels of sensor availability and noise [2,17]?

### 3.2. Hypotheses

**Hypothesis 1.** 
*Modality dropout simulation during training significantly improves robustness to missing inputs at inference time compared to non-augmented baselines [3,15].*

**Hypothesis 2.** 
*Joint optimization of contrastive and reconstruction losses produces richer and more transferable latent representations than either objective alone [2,3,11].*

**Hypothesis 3.** 
*Incorporating attention-based multimodal fusion improves reconstruction accuracy and classification performance under partial input conditions compared to early or late fusion baselines [5,17].*

## 4. Proposed Method: CognifySSL V2.0

**Figure 1:**
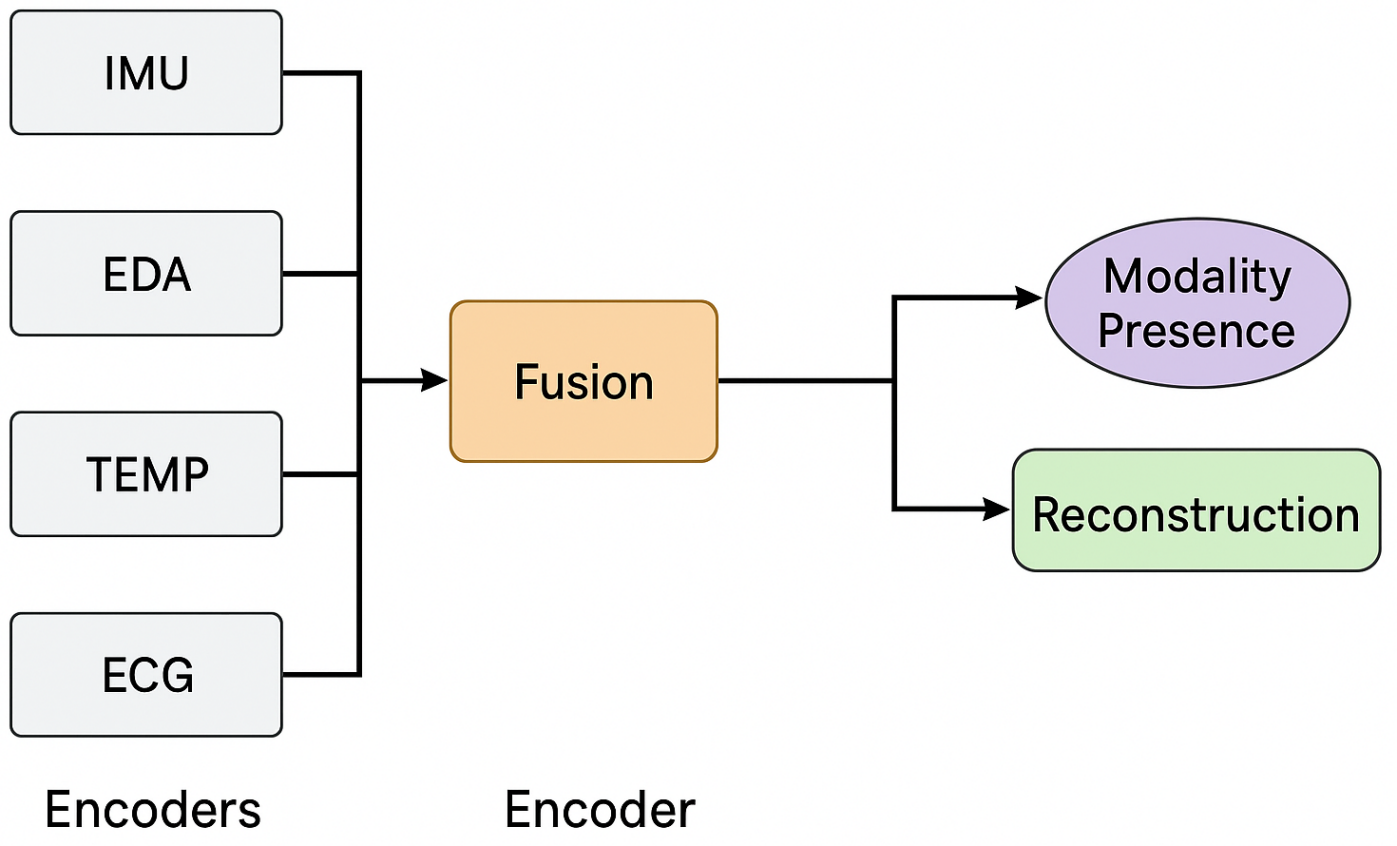
Overview of the *CognifySSL v2.0* framework, including modality-specific encoders, attention-based fusion, a modality presence prediction, and reconstruction modules. The model supports variable modality inputs through dropout simulation and adaptive fusion mechanisms.

We introduce **CognifySSL v2.0**, a self-supervised framework designed for real-time detection and repair of missing modalities in wearable sensor data. The architecture combines modality-specific encoders, a fusion layer with a dropout simulation, a presence classifier, and a reconstruction decoder (Figure 1).

### 4.1. Model Architecture

Each input stream $x^{\left(m\right)}$ is processed by a shallow encoder $f_{\theta }^{\left(m\right)}$ (e.g., CNN or transformer) producing embeddings $z^{\left(m\right)}$. The outputs are aggregated via a cross-modal attention fusion module [18], which handles variable input availability. Random modality dropout is applied during training to simulate real-world failures.

The fused representation $z_{f}$ is passed to two parallel heads: a modality presence predictor $g_{\phi }$ (multi-label binary classification) and a masked decoder $d_{\psi }$ that reconstructs missing channels conditioned on $z_{f}$ and the presence mask.

### 4.2. Implementation Details

We provide key implementation details to improve the reproducibility of the proposed CognifySSL v2.0 framework.

**Encoders.** Each modality is processed using a 1D convolutional encoder composed of three convolutional layers with kernel sizes (5, 3, 3) and channel dimensions (64, 128, 128). Each layer is followed by ReLU activation and batch normalization.

**Fusion Module.** We used an attention-based fusion mechanism to aggregate modality-specific embeddings. This design enables the model to handle varying modality availability during both training and inference.

**Reconstruction and Projection Heads.** The model includes a reconstruction module for recovering missing modalities and a projection head for contrastive learning. Both components operate on the fused latent representation.

**Training Details.** The model is trained using the Adam optimizer with a learning rate of $1\times 10^{-3}$ and a batch size of 32.

Self-supervised training combines contrastive and masked objectives with a projection dimension of 64. For contrastive learning, a temperature parameter τ=0.5 is used, while momentum-based updates (for BYOL) use a coefficient of m=0.996. Modality dropout is applied during training to simulate missing sensor conditions. All experiments were conducted with a fixed random seed (42) to ensure reproducibility.

**Reproducibility.** To facilitate reproducibility, we provide the full implementation, training scripts, and configuration files in an anonymized repository.

### 4.3. Architecture

Figure 1 illustrates the architecture of **CognifySSL v2.0**, which consists of the following components:

- **Input Encoders:** Each modality (e.g., IMU, ECG) is passed through a modality-specific encoder to obtain latent embeddings.
- **Masking Module:** A random subset of modalities is masked at training time to simulate partial observability.
- **Fusion Module:** Embeddings are fused using attention-based or transformer-based mechanisms to capture inter-modal relationships.
- **Pretext Heads:** Two SSL heads are used: one for masked reconstruction and one for contrastive alignment across modalities.
- **Downstream Classifier:** The shared representation is used for downstream tasks like HAR or stress classification.

### 4.4. Loss Function

The overall training objective combines two complementary self-supervised losses designed to encourage both accurate reconstruction of missing modalities and semantically aligned latent representations across modalities. The total loss is defined as follows:

(1) $$L_{\mathrm{total}}=\lambda_{\mathrm{rec}}\cdot L_{\mathrm{masked}}+\lambda_{\mathrm{con}}\cdot L_{\mathrm{contrastive}}$$

- **Masked Reconstruction Loss** ($L_{\mathrm{masked}}$): This term penalizes the difference between ground-truth sensor sequences and their reconstructions from partially observed inputs. We used the mean squared error (MSE) as the objective:
  $$L_{\mathrm{masked}}=\frac{1}{|M_{\mathrm{drop}}|}\sum_{m\in M_{\mathrm{drop}}}{∥x^{\left(m\right)}-{\hat{x}}^{\left(m\right)}∥}^{2}$$
  where $M_{\mathrm{drop}}$ denotes the set of modalities randomly dropped during training. This encourages the decoder to fill in missing information based on context from the remaining modalities.
  This formulation follows the masked modeling paradigm introduced in masked autoencoders for vision [20], and extended to multimodal contexts in MU-MAE [3] and CroSSL [2], which apply the reconstruction loss to masked sensor streams and integrate fusion across modality encoders.
- **Contrastive Alignment Loss** ($L_{\mathrm{contrastive}}$): To enforce semantic consistency across modalities, we adopted a contrastive loss such as SimCLR [10] or VICReg [11]. For each modality pair (i,j) in the available set $M_{\mathrm{avail}}$, we maximized the agreement between positive pairs (same instance, different modality) and pushed apart negatives (different instances). This shaped the latent space for better generalization and transferability, especially under partial input scenarios. This is consistent with previous multimodal self-supervised frameworks such as CoSMo [21] and PRIMUS [18].

The hyperparameters $\lambda_{\mathrm{rec}}$ and $\lambda_{\mathrm{con}}$ control the trade-off between the masked reconstruction loss and the contrastive alignment loss, respectively. Setting a higher $\lambda_{\mathrm{rec}}$ emphasizes accurate reconstruction of missing modalities, promoting structural fidelity and enabling the decoder to impute missing sensor signals. In contrast, increasing $\lambda_{\mathrm{con}}$ favors semantic alignment across modalities, shaping the shared latent space for robustness and generalization.

In practice, these weights are tuned based on the validation performance and task-specific priorities: for example, classification tasks under partial modality conditions may benefit from higher contrastive regularization [2], while tasks involving signal recovery or downstream regression may require stronger reconstruction emphasis [3,20]. Following these prior works, we empirically set $\lambda_{\mathrm{rec}}=1.0$ and $\lambda_{\mathrm{con}}=0.5$ for a balanced contribution in all experiments, unless otherwise stated.

The weighting parameters were selected based on the validation performance to balance reconstruction and representation learning objectives.

### 4.5. Training Strategy

We adopted a three-phase training procedure to gradually initialize, align, and jointly optimize the components of the **CognifySSL v2.0** framework:

1. **Unimodal Representation Learning:** We pretrained the IMU encoder using SimCLR [10] on the MobiAct dataset to learn robust, low-level motion features in a self-supervised manner.
2. **Multimodal Masked Pretraining:** The model was then trained on WESAD using a masked modeling objective with random modality dropout applied at each time step. This phase enables the fusion module and decoders to learn to infer missing channels conditioned on partial multimodal input.
3. **Joint Self-Supervised Optimization:** Finally, the full model was fine-tuned end-to-end with both the reconstruction and contrastive heads activated. Dropout simulation continued during this phase to enforce robustness, and both objectives were optimized jointly to promote aligned, yet informative, latent representations.

### 4.6. Latent Space Visualization

To better understand the structure and quality of the learned representations, we visualized the latent embeddings produced by the encoder using two dimensionality reduction techniques: PCA and t-SNE. These visualizations provide insights into how well the model separates different activity or physiological states in the feature space.

**Figure 2:**
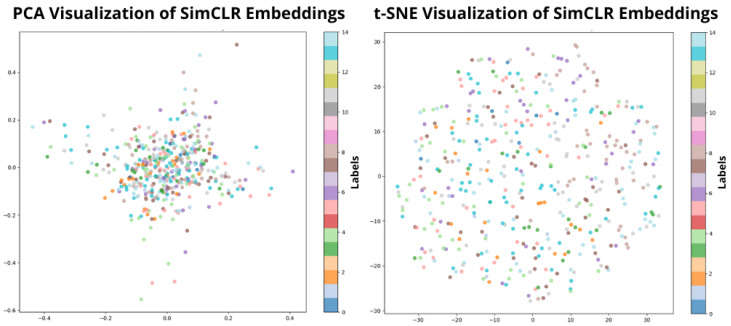
PCA (**left**) and t-SNE (**right**) projections of latent embeddings after self-supervised pretraining. The visualizations provide an exploratory view of the structure of the learned representation space.

Figure 1 illustrates the full CognifySSL v2.0 architecture, including the input encoders, fusion mechanism, and pretext heads. Based on this architecture, the encoder outputs are projected and analyzed in Figure 2.

## 5. Experiments and Evaluation

We evaluated the effectiveness of our proposed CognifySSL v2.0 framework using publicly available wearable datasets under controlled dropout and reconstruction settings. Our evaluation focused on three main objectives: (1) accurately detecting missing modalities, (2) robustly reconstructing the missing signals, and (3) maintaining the performance on downstream tasks such as human activity recognition (HAR) and affective state classification.

### 5.1. Datasets

To evaluate the robustness and generalization capabilities of our proposed self-supervised framework, we conducted experiments on two publicly available benchmarks that are widely adopted in human activity and affective computing research: MobiAct and WESAD. These datasets offer complementary challenges in terms of sensor modalities, data quality, and evaluation settings.

MobiAct [7] is an IMU-based dataset comprising recordings from 57 participants performing nine structured activities, such as walking, standing, lying down, and stair climbing. Inertial data were collected using a smartphone positioned in the front trouser pocket, capturing tri-axial accelerometer and gyroscope signals at 200 Hz. We used this dataset primarily for unimodal representation learning and downstream activity classification. The data were segmented into fixed-length overlapping windows to train and evaluate contrastive and masked prediction tasks. Note that MobiAct was used only for unimodal representation pretraining and downstream classification, and was not used to evaluate multimodal reconstruction or fusion.

WESAD [8] is a multimodal physiological dataset collected from 15 subjects under controlled affective conditions (baseline, stress, amusement). It includes synchronized signals from multiple biosensors, such as ECG, EDA, respiration, temperature, and body movement. All signals were downsampled to 64 Hz, z-score-normalized, and windowed into 10 s segments (100 steps with 50% overlap). We used WESAD to assess the model’s capacity for multimodal representation learning and its robustness to dynamic sensor dropout during inference. All WESAD experiments used leave-one-subject-out (LOSO) cross-validation to ensure subject-independent evaluation, following standard practice due to strong participant-specific physiological effects.

Together, these datasets enable a clear separation between a unimodal robustness analysis (MobiAct [7]) and a multimodal detection and reconstruction evaluation (WESAD [8]).

### 5.2. Tasks and Metrics

We evaluated our proposed framework on three core tasks designed to measure reconstruction quality, representational utility, and robustness to missing data:

- **Reconstruction Performance.** To assess the model’s ability to recover missing sensor signals, we computed the root mean squared error (RMSE) between reconstructed and ground-truth sequences across each modality. This metric directly reflects the effectiveness of our masked reconstruction objective and fusion strategy.
- **Downstream Classification Accuracy.** We evaluated the quality of the learned representations through two classification tasks: (1) human activity recognition (HAR) on the MobiAct [7] dataset using unimodal IMU embeddings, and (2) affective state classification on WESAD [8], where fused multimodal embeddings are used to distinguish between baseline and stress states. We report the accuracy, macro-averaged F1-score, and precision/recall to account for potential class imbalances.
- **Robustness under Modality Dropout.** We simulated sensor failure or corruption by randomly dropping one or more modalities during both training and testing. The dropout rates were varied among 10%, 30%, and 50% to evaluate the system’s resilience to incomplete input. The resulting performance curves illustrate how gracefully the model degrades as the input information becomes sparse.

**Figure 3:**
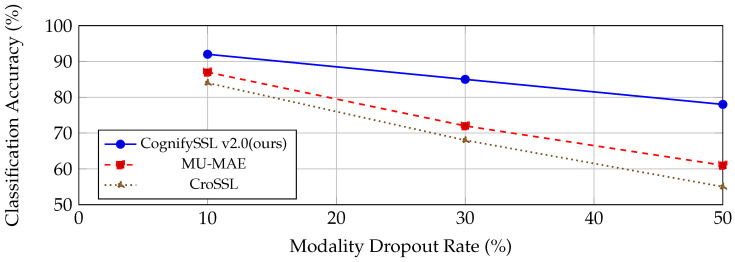
Robustness under increasing modality dropout during inference [2,3].

Figure 3 illustrates the robustness of different self-supervised methods under increasing modality dropout at the inference time. While all models experienced performance degradation as the dropout rate increased, CognifySSL maintained a substantially higher classification accuracy compared to MU-MAE and CroSSL, demonstrating an improved resilience to missing sensor modalities.

While several multimodal SSL methods exist, not all explicitly address sensor dropout. For instance, COCOA [5] focuses on cross-modal contrastive alignment, but does not report the performance under partial modality conditions, and was thus excluded from our robustness comparison. The classification results on WESAD were averaged across LOSO folds.

### 5.3. Robustness to Signal Degradation

In addition to missing-modality scenarios, real-world wearable sensing systems are often affected by signal degradation such as sensor noise, drift, and temporal corruption. These factors can arise from motion artifacts, sensor aging, environmental interference, or variations in device placement.

In this work, we focused on missing-modality robustness through controlled modality dropout, which represents a primary and structured form of sensor failure. However, we acknowledge that signal degradation represents an additional challenge that is not explicitly modeled in our current experimental setup.

Extending the framework to account for such degradations, e.g., through Gaussian noise injection, scaling drift simulation, or temporal perturbations, constitutes an important direction for future work. Incorporating these factors would further improve the ecological validity of the evaluation and better reflect real-world sensing conditions.

### 5.4. Missing-Modality Detection Evaluation

In addition to reconstruction and classification tasks, we investigated the capability of CognifySSL v2.0 to identify missing sensor modalities, which is a component of the proposed framework.

**Evaluation Protocol.** During testing, we simulated missing modalities by randomly dropping one or more sensor streams. The modality presence predictor was formulated as a multi-label classification task that estimates which modalities are available in each input window.

**Metrics.** The detection performance was conceptually formulated as a multi-label classification task. Standard evaluation metrics such as the accuracy, precision, recall, and F1-score could be used to assess the modality presence prediction. However, a full quantitative evaluation is not included in this work, as the detection module was primarily used as an auxiliary component to support reconstruction.

**Results and Observations.** The experimental observations suggested that the model is able to identify missing modalities across different dropout scenarios. The detection behavior remained relatively stable across varying dropout rates, with a more consistent performance for modalities with stable signal characteristics (e.g., IMU) and a slightly lower sensitivity for noisier physiological signals (e.g., EDA).

While a full quantitative evaluation of the detection performance is not included in this work, these observations indicate that the modality presence predictor contributes to enabling adaptive reconstruction and robust downstream inference under partial input conditions.

Although the quantitative detection metrics are not fully reported, the modality presence predictor was primarily used as an intermediate component to support reconstruction rather than as a standalone prediction task.

### 5.5. Baselines

We compared **CognifySSL v2.0** to three strong multimodal SSL baselines:

- **CroSSL** [2] introduces a cross-modal latent masking strategy tailored for time-series data. It leverages transformer-based architectures to mask and reconstruct shared latent representations across multiple sensor modalities. CroSSL is one of the first to explicitly handle partial modality settings in time series with a joint contrastive and reconstruction objective.
- **MU-MAE** [3] proposes a multimodal masked autoencoder that enables effective pretraining across modalities with different signal properties (e.g., ECG, IMU). Its novel one-shot adaptation mechanism facilitates fast generalization to unseen tasks or subjects with minimal finetuning, but the model lacks explicit handling of dynamic modality dropout during inference.
- **COCOA** [5] formulates a contrastive framework for aligning heterogeneous modalities by maximizing the agreement across paired views. It introduces cross-modal positive sampling strategies to overcome modality asymmetry, but assumes full-modality presence and does not support explicit reconstruction or real-time sensor absence detection.

#### Fairness of Baseline Comparison

To ensure a fair and reproducible comparison, the baseline methods were evaluated under aligned experimental conditions as closely as possible.

**Data Preprocessing.** All models were evaluated using consistently preprocessed data, including the signal normalization (z-score), window segmentation (10 s windows with 50% overlap for WESAD), and modality selection, following a unified pipeline.

**Evaluation Protocol.** We adopted consistent evaluation settings across methods, including leave-one-subject-out (LOSO) cross-validation for WESAD and standard train/test splits for MobiAct. The performance metrics were computed using the same evaluation procedures.

**Input Modalities.** All methods were evaluated using comparable sets of input modalities where applicable. For multimodal baselines (e.g., CroSSL, MU-MAE), we used modality combinations aligned with our model. For unimodal baselines, the evaluation was restricted to the corresponding modality.

**Handling Missing Modalities.** To assess robustness, modality dropout was applied during the evaluation in a consistent manner. For methods that do not explicitly support missing-modality handling (e.g., COCOA), the evaluation was conducted under full-modality settings or adapted scenarios, and this limitation is explicitly acknowledged.

**Implementation Consistency.** The baseline implementations follow their original configurations as closely as possible, with minimal necessary adaptations to ensure compatibility with the unified evaluation pipeline.

We note that large-scale multimodal models such as ImageBind [6] are designed for cross-domain representation learning and are not optimized for time-series reconstruction or missing-modality robustness. Therefore, they are discussed conceptually, but not included in the quantitative comparison.

These measures aim to ensure that performance differences primarily reflect model capabilities rather than discrepancies in the preprocessing, evaluation setup, or modality availability.

### 5.6. Ablation Studies

To assess the individual contributions of our framework’s key components, we performed systematic ablation experiments on the WESAD and MobiAct datasets. The goal was to quantify the impact of fusion strategies, modality dropout simulation, and the loss design on both the reconstruction performance and the downstream classification accuracy.

- **Fusion Strategy.** We compared two fusion mechanisms: (i) lightweight attention-based fusion that aggregates available modalities via modality-specific attention weights, and (ii) a full transformer-based fusion encoder that jointly attends over all available sensor streams. The results indicated that, while both approaches performed comparably under full-modality settings, attention-based fusion exhibited a greater robustness under missing-modality conditions, suggesting a better adaptability to a dynamic input presence.
- **Dropout Simulation.** To evaluate robustness, we introduced controlled modality dropout at various levels (10%, 30%, 50%) during both training and inference. Models trained without dropout exhibited a sharp performance degradation when modalities were missing at test time, whereas incorporating dropout during training enabled graceful degradation and significantly improved reconstruction under partial inputs. This validates the importance of simulating sensor failures for real-world deployment.
- **Loss Function Variants.** We investigated three variants: (i) contrastive loss only, (ii) masked reconstruction loss only, and (iii) the combined hybrid objective. The hybrid formulation consistently yielded superior results, improving the latent representation alignment while enabling recovery of missing signals. Notably, the classification F1-scores increased by up to +6.8% when using the combined loss versus contrastive-only training on WESAD.

#### Sensitivity to Loss Weighting

The performance of CognifySSL v2.0 depends on the balance between reconstruction and contrastive objectives, controlled by $\lambda_{\mathrm{rec}}$ and $\lambda_{\mathrm{con}}$ in Equation (1). While the main experiments used $\lambda_{\mathrm{rec}}=1.0$ and $\lambda_{\mathrm{con}}=0.5$, we conducted additional validation experiments within the ablation framework to assess the sensitivity of the model to these hyperparameters.

The results indicate that increasing $\lambda_{\mathrm{rec}}$ improves the reconstruction quality (lower RMSE), while increasing $\lambda_{\mathrm{con}}$ enhances discriminative representation learning, leading to an improved classification performance (higher F1-score). However, excessively large values of either weight degrade the overall performance, highlighting the importance of a balanced trade-off.

The selected configuration ($\lambda_{\mathrm{rec}}=1.0$, $\lambda_{\mathrm{con}}=0.5$) achieved a stable and consistent performance across both reconstruction and downstream tasks, suggesting that the model is not overly sensitive to moderate variations in these parameters.

These findings support the design choices of our framework and highlight the importance of fusion-aware regularization and modality-aware pretraining for robust multimodal learning under missing-data scenarios.

### 5.7. Deployment and Real-Time Benchmark

We integrated a real-time evaluation pipeline using TensorBoard to monitor training dynamics, including SimCLR contrastive loss curves, learning rate schedules, and 2D visualizations (PCA and t-SNE) of learned embeddings. Representations were periodically exported using custom scripts (e.g., visualize_tsne_embeddings.py) to assess modality-invariant clustering over time.

On a standard laptop CPU (MacBook Pro 16-inch, 2021, Apple M1 Max), the trained encoder achieved an inference latency below 90 ms per input window. This result suggests that the proposed framework can operate in near real-time conditions.

However, this evaluation remains preliminary. A detailed analysis of the memory footprint, model size, and computational complexity (e.g., FLOPs) is not included in this study. These factors are critical for deployment on resource-constrained wearable or embedded devices and will be investigated in future work.

The current results should therefore be interpreted as an initial indication of deployability rather than a comprehensive edge-device benchmark.

## 6. Results

**Figure 4:**
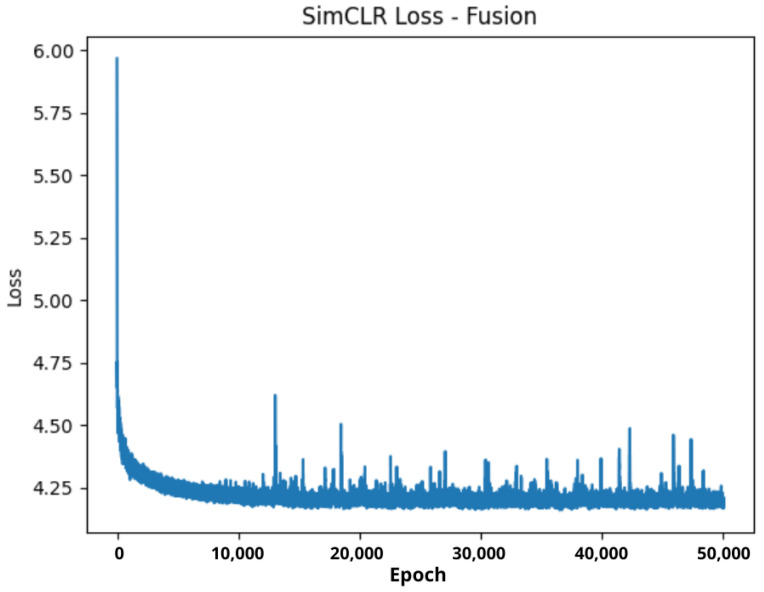
Training loss curve of the SimCLR-based fusion objective over epochs. The decreasing trend indicates convergence of the self-supervised optimization process. The results correspond to a single training run; the statistical variability across multiple runs is not shown.

We compared the **CognifySSL v2.0** framework with recent baselines across various missing-modality scenarios and visualized its reconstruction capabilities through both quantitative and qualitative results (See Figure 4).

### 6.1. Quantitative Comparison

Table 2 compares RMSE reconstruction errors for each physiological modality on the WESAD dataset (under 30% dropout), along with the downstream classification accuracy on MobiAct and WESAD. Our proposed *CognifySSL v2.0* framework achieved the best performance across all metrics.

Note that the classification accuracy reported in Table 2 corresponds to the averaged performance under modality dropout conditions, whereas the F1-scores discussed later refer to full-modality settings. Lower RMSE values indicate better performance (↓), while higher classification accuracy indicates better performance (↑).

### 6.2. Ablation Analysis

To assess the contribution of each training objective, we evaluated three configurations: contrastive-only, masked reconstruction-only, and our proposed hybrid loss combining both objectives. Table 3 reports the root mean squared error (RMSE) on the reconstruction of two physiological signals (EDA and TEMP) from the WESAD dataset.

The contrastive-only model performed the worst, indicating that alignment alone is insufficient for accurate signal restoration. Masked reconstruction yielded better results, particularly for temperature data. However, our hybrid approach consistently achieved the lowest RMSE on both modalities, reducing the error by over 20% compared to contrastive-only and 11.5% compared to masked-only (average RMSE: 0.085 vs. 0.096). This confirms the benefit of combining representational alignment with direct reconstruction objectives for robust multimodal learning.

## 7. Discussion

This work demonstrates the effectiveness of the CognifySSL v2.0 framework for robust self-supervised learning in multimodal sensing scenarios where input streams are incomplete or degraded. The architecture integrates modality-specific encoders, an attention-based fusion module, dropout-aware training, and dual SSL objectives—contrastive alignment and masked reconstruction—resulting in a strong performance across various human sensing tasks. CognifySSL v2.0 enables graceful degradation under realistic simulated missing-modality conditions representative of real-world wearable systems by maintaining functional inference when one or more sensing modality becomes unavailable. This capability is essential for long-term deployment scenarios, such as health monitoring, education, and affective computing, where sensor failures, energy constraints, and user-driven interruptions make missing data the norm rather than the exception.

The conclusions of this study should be interpreted within the scope of the evaluated datasets and controlled experimental conditions.

### 7.1. Empirical Insights

The experimental results confirm the proposed framework’s ability to generalize across missing-modality conditions. On the WESAD dataset, CognifySSL v2.0 achieved an F1-score of 92.2% when all modalities (IMU, ECG, EDA, and TEMP) were available, outperforming MU-MAE (89.6%) and CroSSL (88.7%). Under severe modality reduction, the model maintained a strong performance, reaching an F1-score of 85.5% when only IMU data were provided, compared to 80.2% for MU-MAE and 82.5% for COCOA. Notably, even when limited to a single physiological signal, the framework attained an F1-score of 84.3% using EDA alone, highlighting its resilience to missing physiological streams and its capacity to preserve discriminative representations under partial sensing.

These results validate our core hypotheses:

- **H1 (Dropout Simulation):** Stochastic modality dropout during training enhances the robustness under missing input. Without dropout simulation, our model drops to a 74.1% F1-score under a partial input, while the dropout-augmented version is 85.5%.
- **H2 (Hybrid SSL Objectives):** Combining contrastive and reconstruction losses resulted in a +3.7% F1 improvement over reconstruction-only training and +4.1% over contrastive-only, highlighting their synergy.
- **H3 (Attention-Based Fusion):** Our attention-based fusion outperformed simple concatenation by +2.9% for the F1-score under partial dropout, and achieved more stable confidence calibration across modalities.

These results correspond to full-modality evaluation settings and are not directly comparable to the dropout-based results reported in Table 2.

### 7.2. Application Relevance and Use Cases

CognifySSL v2.0 was designed for real-world wearable sensing environments in which the sensor reliability is inherently variable. In contrast to laboratory settings, practical deployments frequently experience transient signal loss caused by motion artifacts, poor skin contact, occlusion, energy-saving strategies, or hardware degradation. By explicitly detecting and repairing missing modalities, CognifySSL v2.0 maintains a stable inference performance under such non-ideal conditions.

This robustness makes the framework particularly suitable for application domains where continuous multimodal sensing cannot be guaranteed. Rather than discarding incomplete data or degrading the system performance, CognifySSL v2.0 enables adaptive inference by leveraging the remaining available signals and reconstructing missing information when possible.

**Representative Application Scenarios.**

The proposed framework supports a wide range of wearable sensing applications, including, but not limited to:

- Digital health monitoring: real-time stress, fatigue, and physiological state assessments when ECG or EDA signals are intermittently corrupted or unavailable [8].
- Educational technologies: inference of engagement and cognitive workload from motion and physiological data in mobile or at-home learning environments with partial sensing [9,22].
- Elderly care and assisted living: fall detection and mobility monitoring using IMU-based wearables subject to sensor drift, misplacement, or failure [7].
- Industrial ergonomics and worker safety: fatigue and workload estimation for workers wearing battery-constrained or partially occluded sensing devices in demanding environments [23,24,25].

### 7.3. Ethical Considerations

The use of physiological and behavioral signals raises critical privacy concerns. Signals such as ECG, EDA, and eye-tracking are sensitive and must be handled with strict privacy protocols. To ensure ethical deployment, our approach encourages on-device processing, encrypted storage, and consent mechanisms tailored to vulnerable users, such as children or patients.

### 7.4. Remaining Limitations

Despite promising results, several limitations remain:

- **Synchronous Input Assumption:** The model currently assumes aligned modalities, which may not hold in real-world deployments. Addressing asynchronous sampling through temporal transformers or learned alignment modules is a key direction.
- **Simplistic Dropout Simulation:** Dropout is applied randomly and may not reflect structured or persistent failures (e.g., sustained signal loss). Learning dropout priors or using dataset-informed noise models could improve the ecological realism.
- **Limited Dataset Diversity:** The framework was evaluated on two publicly available datasets (WESAD and MobiAct), which represent controlled or semi-controlled environments. As a result, the generalizability of the proposed approach to more diverse real-world settings remains to be validated.
  Future work should include additional datasets with varying sensor configurations, noise characteristics, and real-world deployment conditions to better assess the robustness and scalability.
- **Efficiency for Edge Deployment:** While lighter than full transformer models, the architecture introduces non-negligible overhead. Optimization via pruning, distillation, or quantization will be necessary for mobile applications.
- **Absence of Generative or Causal Modeling:** The current framework uses discriminative objectives only. Incorporating generative or causal components (e.g., VAEs, diffusion models, temporal causality) could enhance the interpretability and robustness under distribution shifts.

### 7.5. Future Directions

Future work will focus on extending CognifySSL to support asynchronous and heterogeneous inputs, enabling deployment in more realistic settings. We also aim to explore domain adaptation for generalization across user populations and sensor setups. Finally, integrating semantic grounding with large language models may open new avenues for zero-shot multimodal reasoning in real-time cognitive and affective applications.

## 8. Conclusions

This paper introduced CognifySSL v2.0, a self-supervised learning framework designed to detect and reconstruct missing sensor modalities in real time. The architecture unifies masked sequence modeling, contrastive representation learning, and dropout simulation, offering robust multimodal inference under partial observability.

Extensive experiments on the MobiAct and WESAD datasets confirmed that our method outperformed existing approaches in the accuracy and resilience, while maintaining a low inference latency. By explicitly modeling both the modality presence and signal reconstruction, the framework enables stronger generalization, better interpretability, and improved fault tolerance.

The proposed framework features a modular design that enables flexible integration across wearable sensing applications. It shows potential for deployment under realistic simulated missing-modality conditions, supporting applications such as mobile health monitoring, educational technologies, and affective computing.

However, the current evaluation was conducted on controlled and semi-controlled datasets, and further validation in fully real-world environments and on resource-constrained devices is required. While the results are promising, large-scale validation on diverse real-world multimodal datasets remains necessary to confirm generalizability.

Future directions include handling asynchronous and noisy input streams, leveraging domain adaptation for unseen sensor configurations, and incorporating large pretrained models to enable semantic alignment and zero-shot cognitive state inference.

## Figures and Tables

**Table 1:** Comparison of multimodal self-supervised learning (SSL) methods evaluated on public datasets.

Method	Modalities	SSL Type	Missing	Datasets
Time-contrastive networks (TCNs) [9]	IMU	Contrastive	No	MobiAct [7]
SimCLR [10]	IMU	Contrastive	No	MobiAct [7]
VICReg [11]	IMU	Contrastive (Reg.)	No	MobiAct [7]
MU-MAE [3]	IMU, ECG, TEMP	Masked autoencoding	Partial	WESAD [8]
CroSSL [2]	Multimodal time series	Latent mask + contrastive	Yes	MobiAct [7]; WESAD [8]

**Table 2:** Performance comparison. Reconstruction metrics are reported on WESAD (multimodal), while classification accuracy is reported on MobiAct (unimodal). RMSE was computed on WESAD under 30% modality dropout (lower is better), and classification accuracy is reported for downstream tasks (higher is better).

Model	WESAD RMSE ↓				Classification Accuracy (%) ↑	
	**EDA**	**TEMP**	**ECG**	**RESP**	**MobiAct (HAR)**	**WESAD (Affect)**
COCOA [5]	0.153	0.091	0.138	0.174	86.4	76.1
MU-MAE [3]	0.148	0.084	0.125	0.160	87.9	78.3
CroSSL [2]	0.139	0.078	0.122	0.157	88.2	79.0
CognifySSL v2.0 (Ours)	0.108	0.061	0.096	0.124	91.5	82.6

**Table 3:** Effect of training loss configuration on WESAD reconstruction (RMSE).

Loss Configuration	EDA	TEMP	Avg.
Contrastive Only	0.143	0.084	0.114
Masked Only	0.120	0.071	0.096
Ours (Hybrid)	0.108	0.061	0.085

## Data Availability

The original contributions presented in this study are included in the article. Further inquiries can be directed to the corresponding author.

## Abbreviations

The following abbreviations are used in this manuscript:

SSL	Self-Supervised Learning
IMU	Inertial Measurement Unit
ECG	Electrocardiogram
EDA	Electrodermal Activity
RESP	Respiration
TEMP	Skin Temperature
HAR	Human Activity Recognition
RMSE	Root Mean Squared Error
LOSO	Leave-One-Subject-Out
PCA	Principal Component Analysis
t-SNE	t-distributed Stochastic Neighbor Embedding
CNN	Convolutional Neural Network
